# The Future of Regulatory T Cell Therapy: Promises and Challenges of Implementing CAR Technology

**DOI:** 10.3389/fimmu.2020.01608

**Published:** 2020-07-24

**Authors:** Yasmin R. Mohseni, Sim L. Tung, Caroline Dudreuilh, Robert I. Lechler, Gilbert O. Fruhwirth, Giovanna Lombardi

**Affiliations:** ^1^Peter Gorer Department of Immunobiology, MRC Centre for Transplantation, School of Immunology and Microbial Science, King's College London (KCL), Guy's Hospital, London, United Kingdom; ^2^Imaging Therapies and Cancer Group, Department of Imaging Chemistry and Biology, School of Biomedical Engineering and Imaging Sciences, King's College London, London, United Kingdom

**Keywords:** Tregs (regulatory T cells), transplantation, CAR (chimeric antigen receptor), cell therapy, autoimmunity, regulatory, antigen specific

## Abstract

Cell therapy with polyclonal regulatory T cells (Tregs) has been translated into the clinic and is currently being tested in transplant recipients and patients suffering from autoimmune diseases. Moreover, building on animal models, it has been widely reported that antigen-specific Tregs are functionally superior to polyclonal Tregs. Among various options to confer target specificity to Tregs, genetic engineering is a particularly timely one as has been demonstrated in the treatment of hematological malignancies where it is in routine clinical use. Genetic engineering can be exploited to express chimeric antigen receptors (CAR) in Tregs, and this has been successfully demonstrated to be robust in preclinical studies across various animal disease models. However, there are several caveats and a number of strategies should be considered to further improve on targeting, efficacy and to understand the *in vivo* distribution and fate of CAR-Tregs. Here, we review the differing approaches to confer antigen specificity to Tregs with emphasis on CAR-Tregs. This includes an overview and discussion of the various approaches to improve CAR-Treg specificity and therapeutic efficacy as well as addressing potential safety concerns. We also discuss different imaging approaches to understand the *in vivo* biodistribution of administered Tregs. Preclinical research as well as suitability of methodologies for clinical translation are discussed.

## Introduction

Regulatory T cells (Tregs) are a subset of T cells that function to maintain homeostasis and prevent autoimmunity (1). Tregs make up 5–10% of the CD4^+^ T cell population (2) and are characterized by co-expression of CD4, CD25, the transcription factor Forkhead box protein 3 (FOXP3) and low levels of CD127. Although conventional human T cells (Tconv) can transiently express FOXP3, high FOXP3 levels and demethylation of the Treg specific demethylated region (TSDR), a conserved region within the *FOXP3* gene, are distinct features of Tregs (3). The importance of FOXP3 in Tregs is supported by the evidence that mutations in the *FOXP3* locus lead to Treg dysfunction and severe autoimmunity, as was first identified in *Scurfy* mutant mice (4) and the immunodysregulation polyendocrinopathy enteropathy X-linked syndrome (IPEX) in humans (5).

Tregs are divided in thymus-derived (tTregs) and peripheral-derived Tregs (pTregs) (6). During T cell development, those naïve CD4^+^ T cells receiving an intermediate TCR signal are driven to differentiate into Tregs—the quantitative difference in strength of such signal is thought to determine Tconv cell or Treg lineage commitment (7). Peripheral Tregs develop when FOXP3^−^ Tconv encounter repeated stimulation to non-self antigens or receive inadequate co-stimulation, as well as exposure to IL-10 and TGF-β (8).

Tregs suppress the immune system by different mechanisms including contact-dependent mechanisms, through CTLA-4 engagement for example, and contact-independent, such as the release of cytokines e.g., IL-35 or IL-10 [reviewed in (9)]. Given their proven role in preventing autoimmune diseases, Tregs have obvious potential in the promotion of tolerance. Although human Tregs constitute a small proportion of circulating CD4^+^ T cells, they are attractive candidates for immunotherapeutic purposes given that they can be isolated, manipulated and expanded in large numbers *in vitro*. Tregs can be applied in the treatment of autoimmune diseases and in the prevention of transplant rejection and graft vs. host disease (GvHD).

## Adoptive Treg Therapy: From Polyclonal to Antigen Specific

The first phase I clinical trials investigating the safety of adoptive transfer of Tregs were in the treatment of bone marrow patients to prevent GvHD, NCT00602693 (10–12). These trials demonstrated the safety and efficacy of Treg therapy. Autologous polyclonal Tregs have been infused in patients with type 1 diabetes (T1D) as well, demonstrating again the safety and feasibility of adoptive Treg therapy in this disease setting [ISRCTN06128462, (13) and NCT02691247, (14)]. Treg therapy has reached the organ transplant arena as well (UMIN-000015789 and NCT02088931) (15, 16). We have demonstrated the safety of adoptively transferred Tregs in two phase I clinical trials in liver (ThRIL, NCT02166177) and kidney (ONE study, NCT02129881) transplant patients (17, 18).

However, whilst the above clinical studies have shown the potential of polyclonally expanded Tregs, we and others have demonstrated the superiority of antigen-specific Tregs compared to polyclonal Tregs in animal models. Tang et al. successfully isolated and expanded Tregs from a transgenic mouse expressing a TCR specific for an islet antigen, and showed that antigen-specific Tregs prevented and even reversed diabetes in non-obese diabetic mice (19, 20). More recently, human Tregs were modified *in vitro* to generate Tregs specific for donor antigens, by co-culturing Tregs with donor-derived dendritic cells (DCs) or B cells (21, 22). The superiority of donor-specific human Tregs compared to polyclonal Tregs was demonstrated *in vitro* and *in vivo* in a humanized mouse model of human skin transplant (21, 22). Similar results were obtained *in vitro* by Zheng et al. as they demonstrated that mature B cells were better stimulants than immature DCs in generating Tregs expressing higher levels of FOXP3 and CD25, and with superior suppressive capacity (23). Already as part of the ONE Study (NCT02129881) kidney transplant patients have been treated with donor-specific Tregs and additional clinical trials in transplant patients are investigating the use of donor-reactive Tregs [reviewed by (9)].

Evolving from the use of APCs to generate Tregs with specificity for the target antigen, research has shifted toward gene transfer. Wright et al. transduced Tregs with a TCR specific for ovalbumin (OVA) and restricted by the MHC-class II A^b^. These Tregs transferred *in vivo* were able to inhibit a well-established antigen induced arthritis in which mice were immunized with methylated BSA (mBSA) followed by intra-articular knee re-challenge with mBSA to induce T cell-mediated tissue damage. The OVA-specific Tregs were able to decrease inflammation to the knee but only when OVA was present (24). In the same study a similar effect was obtained with CD4^+^ Tconv transduced with the same TCR and FOXP3; engineering CD4^+^ Tconvs to express FOXP3 endows them with a suppressive function (24). We generated Tregs from C56BL/6 recipient mice specific for donor BALB/c antigen by retroviral transduction of a TCR specific for a peptide derived from MHC-class I K^d^ and presented by MHC-class II A^b^. We demonstrated that Tregs with this specificity contributed to the indefinite survival of BALB/c heart transplants into B6 (25). Brusko et al. transduced human Tregs with a TCR specific for the melanoma antigen tyrosinase and restricted by HLA-A^*^0201. Tregs were expanded *in vitro* and administered *in vivo* in a tumor model. They were able to inhibit effector T cells leading to tumor growth (26). Hull et al. transduced Tregs with two TCRs isolated from islet-specific and influenza-specific CD4^+^ T cell clones. The authors showed that the ability of the islet antigen-specific TCRs to induce Treg mediate antigen-specific suppression *in vitro* was significantly lower when compared to what was achieved using TCRs with specificity for viral antigens (27). More recently, Kim et al. transduced Tregs with a TCR specific for myelin basic protein (MBP) isolated from a T cell clone derived from a multiple sclerosis patient (28). These Tregs suppressed MBP-specific T effector *in vitro* and *in vivo* they ameliorated experimental autoimmune encephalitis (EAE) (28).

As an alternative to the use of Tregs as cell therapy, several studies have looked at generating Tregs by manipulating CD4^+^ Tconv cells to express FOXP3. In hemophilia, up to one third of patients receiving therapeutic factor VIII (FVIII) infusions develop neutralizing antibodies. Herzog et al. transduced CD4^+^ Tconv with FOXP3 and FVIII. Following administration of these cells to hemophilia A mice, the formation of neutralizing antibodies to FVIII was suppressed (29). In an animal model of type 1 diabetes, Jaeckel et al. transduced islet-specific CD4^+^ Tconv with FOXP3. These cells were activated in the pancreatic lymph nodes and reversed recent-onset diabetes (30). Beavis et al. showed that the ectopic expression of FOXP3 in pathogenic synovial T cells from rheumatoid arthritis patients attenuated their function (31). Loser et al. showed the efficacy of FOXP3-transduced Tconv in suppressing contact hypersensitivity responses in mice. Moreover, these cells diminished autoimmune dermatitis in CD40L transgenic mice and cleared antinuclear antibodies (32). These studies are seminal demonstrating the acquisition by Tconvs of a suppressive profile, equally research within immunoregulation has lately been more focused toward enhancing Tregs for cell therapy.

An alternative method to confer specificity to Tregs is by transducing these cells with chimeric antigen receptors (CAR). CAR technology offers some advantages over TCR engineering. These include bypassing HLA restriction upon activation of T cells expressing CARs, increased specificity through the requirement of co-receptor signaling, and the targeting flexibility of CARs (any soluble or surface multivalent antigen can serve as target). In the following sections we focus on CARs to enhance Treg therapy.

## Chimeric Antigen Receptors: Lessons From Cancer Therapy

CARs have been developed and by now clinically implemented in oncology to treat certain cancers. They represent an approach to fine-tune adoptively transferred therapeutic T cells to target specific antigens by-passing MHC-restriction and thereby enable these therapeutic cells to attack the cancer. CARs are artifical molecules engineered into target cells. They are composed of an extracellular target-recognition domain (e.g., a scFv specific for the target antigen), hinge and transmembrane domains, and intracellular signaling domains to propagate activation signals as a consequence of extracellular target engagement. CARs are less sensitive in response than TCRs due in part to the number of molecules involved in the TCR machinery, i.e., CD4/CD8 co-receptors, immunoreceptor tyrosinase-rich activation motifs (ITAMs), and subunits within the receptor complex (CARs require 100–10,000 molecules per target cell while TCRs need <10 molecules per target cell) but bind with higher affinity than the TCR; although studies have investigated increasing CAR sensitivity by incorporating lower affinity single-chain variable fragment [scFv; (33, 34)].

### From CAR-T Cells in Oncology to CAR-Tregs

The first CAR was composed of a CD3ζ chain of the TCR/CD3ζ complex, but T cell activation was neither persistent *in vivo*, nor sustained and the T cells did not proliferate sufficiently (35, 36). Second generation CARs contain an additional intracellular feature, a co-stimulatory domain, which has the purpose to potentiate the signaling response of the CAR. Several co-stimulatory domains including those from CD28, 4-1BB (CD137) and OX40 (CD134) molecules have been explored in CAR-T cell therapy. Third generation CARs are composed of two different co-stimulatory molecules. Indeed it were second generation CARs that led to the breakthrough in cell-based cancer immunotherapy. In 2017, the FDA approved the first clinical products, tisagenlecleucel and axicabtagene ciloleucel—trademarked as Kymriah® and Yescarta—which are autologous CD19b-targeted CAR-T-cell immunotherapies for the treatment of B-cell acute lymphoblastic leukemia and B-cell lymphoma, respectively (37–39). CAR-T immunotherapies have the potential to be curative, but so far not all patients have responded and sometimes the effects were only temporary (39–41). CAR-T cell therapy has been also associated with severe/life-threatening side-effects and fatalities during clinical trials (42, 43). Research into CARs specific for tumor-related antigens in hematological malignancies paved the way for the application of CARs in immunoregulation. CAR-T cells have been already applied to treat autoimmune disease. In an animal model of pemphigus vulgaris, which is a rare severe autoimmune disease in which blisters of varying sizes break out on the skin and mucous membranes, chimeric auto-antibody receptor (CAAR)-T cells were generated with specificity for the keratinocyte adhesion protein Dsg3 (44). The CAAR-T cells exhibited specific cytotoxicity to anti-Dsg B cells *in vivo* without off-target toxicity (44). Although engineering CAAR-T cells may be effective in inhibiting some autoimmune diseases, Tregs can also be applied due to their powerful immunosuppressive and tolerance-promoting properties.

Tregs have been transduced to express CARs and tested in pre-clinical models of autoimmunity, GvHD and transplantation as well as colitis. Elinav et al. generated a transgenic mouse whose T cells including the Tregs expressed a CAR specific for 2,4,6-trinitrophenol. The adoptive transfer of CAR-Tregs to wild-type mice suffering 2,4,6-trinitrobenzenesulfonic acid-induced colitis was associated with significant amelioration of colitis and improved survival (45). The same group generated Tregs expressing a CAR specific for the human carcinoembryonic antigen (CEA). These Tregs markedly suppressed the severity of colitis in the CEA transgenic mouse, CEABAC, where colitis was induced by transfer of effector T cells specific for CEA (46). Another study used a CEA transgenic mouse to show that CEA-specific CAR-Tregs can inhibit allergic airway inflammation (47). More recently, Tenspolde et al. generated CAR-Tregs specific for insulin but despite them proliferating in response to insulin and being suppressive *in vitro*, these CAR-Tregs did not prevent spontaenous diabetes in mice; interestingly these cells persisted up to 4 months post adoptive transfer (48). Furthermore, in a mouse model of hemophilia A, Zhang et al. created Tregs expressing a B cell-targeting antibody receptor (BAR) containing the immunodominant FVIII C2 or A2 domains. The BAR-Tregs completely prevented anti-FVIII antibody development in FVIII-immunized mice. They also demonstrated a direct effect on FVIII-specific B cells (49).

In transplantation, we and others have generated Tregs expressing an HLA-A2-specific CAR (A2-CAR-Tregs) (50–52). We have shown that A2-CAR-Tregs were functionally superior compared to polyclonal Tregs *in vitro* and *in vivo* in a humanized mouse model of BRG mice bearing a human skin transplant reconstituted with 5:1 PBMCs to CAR-Tregs, assessed by histological analysis 5 weeks post adoptive transfer (51). Noyan et al. also demonstrated the efficacy of an A2-CAR-Tregs in inducing indefinite survival of allogeneic human skin transplants in a humanized mouse model of NRG mice injected intraperitoneally with 7.5:1 PBMCs to A2-CAR-Tregs and graft survival was assessed (52). Similar A2^+^CAR-Tregs were also shown to ameliorate xenogeneic GvHD (50). Lately, the Levings group produced a panel of humanized HLA-A2 CAR-Tregs and developed a method to map the specificity of CARs, showing that humanization reduced HLA-A cross-reactivity (53). Recently, the same group also investigated the ability of murine HLA-A2 CAR-Tregs to prevent allograft rejection in immunocompetent mice (54). The results showed that these Tregs prolonged skin allograft survival and humural alloresponses but not in presensitised mice, suggesting HLA-A2 CAR-Tregs are unable to inhibit memory T or B cell responses (54).

In the following sections we review the challenges for CAR-Treg therapy and discuss ways to improve CAR-Treg function, safety and specificity for clinical applications.

### CAR Co-stimulatory Endodomain Functions in T Cells

Past studies have focused on optimizing the CAR co-stimulatory endodomain design to provide robust CAR-T cells for fighting cancer (55) of which a wide variety had been tested. For example, CAR co-stimulatory endodomains tested in T cells in addition to CD28 include 4-1BB, OX40, inducible T cell co-stimulator (ICOS) and CD27. Zhang et al. reported that 4-1BB co-stimulation plays an important role for memory CD8^+^ T cell proliferation *ex vivo* and is superior to CD28 co-stimulation in terms of generating antigen-specific CD8^+^ T cell (56). Transduction of CD4^+^ (57) and of a mixture of CD4^+^ and CD8^+^ (1:1 ratio) (58) T cells with a CAR construct incorporating 4-1BB resulted in augmented T cell longevity. This was due to 4-1BB co-stimulation via the CAR decreasing the exhaustion rate of T cells induced by tonic CAR signaling (57). In another study, Li et al. showed that CAR CD4^+^ and CD8^+^ T cells with 4-1BB co-stimulatory endodomain improved T cell function via the NF-κB signaling pathway. Compared to the CD28 co-stimulatory domain, 4-1BB was more associated with the upregulation of anti-apoptotic proteins, which might explain their function in prolonging T cell longevity (59). Whilst OX40 activity enhanced CD4^+^ and CD8^+^ T cell expansion and survival, it also blocked thymic CD4^+^ Treg activity and antagonized the generation of inducible CD4^+^ Tregs (60–62). However, OX40 activity upregulated anti-apoptotic Bcl-2 family members including Bcl-xL, Bcl-2 and Bfl-l and molecules involved in the cell cycle such as survivin and aurora B kinase (63–65). Additionally, Hombach et al. found that CD4^+^ T cells transduced with a CAR containing an OX40 endodomain abrogated IL-10 secretion, even in conjunction with a CD28 co-stimulation domain, without impairing the other Teff functions, tipping the balance against suppression in cancer (66). Prior to that, the authors investigated the effect of OX40, 4-1BB, and CD28 CAR endodomains in CD4^+^ and CD8^+^ T cells, and determined that CD28 was the most potent at initiating a T cell response, and OX40 and 4-1BB sustained the response with OX40 outperforming the other two for the most prolonged time (67). Another co-stimulatory molecule expressed by T cells is ICOS, which is essential for T cell activation and proliferation (68). ICOS has a significant homology to CD28 and CTLA-4 (69, 70) but is not constitutively expressed on resting T cells but upregulated upon TCR and/or CD28 engagement (69, 71). Guedan et al. demonstrated that ICOS expression via CAR CD4^+^ and CD8^+^ T cells enhanced anti-tumor activity and promoted cell survival longer than 4-1BB or CD28 CAR-T cells (72). CD27 is essential for CD4^+^ T cell functions such as promoting antigen-specific cell expansion of naïve T cells and the generation of memory T cells (73). CD27 co-stimulation via CAR CD4^+^ and CD8^+^ T cells upregulates anti-apoptotic Bcl-XL protein expression and resistance to antigen-induced apoptosis, leading to increased numerical expansion although it underwent equal cell division without CD27 (CD3ζ alone). CD27 CAR-T cells may be better than CD28 CAR-T cells due to enhanced survival and accumulation thus quantitatively increased response (74).

However, whether expression of these co-stimulatory endodomains via CARs on Tregs enhances their function in a similar manner to those found in CAR-T cells is still to be elucidated.

## Clinical Products of CAR-T

Engineering CAR-Tregs destined for the clinic involves different stages in the GMP facility that need to be optimized. Currently, GMP protocols rely on either magnetic isolation of total CD4^+^CD25^+^ Treg populations, or fluorescence-activated cell sorted (FACS) (75). It is advisable that for the generation of CAR-Tregs the Tregs need to be highly pure to avoid any expansion of “contaminating” Teff. Delivering the CAR to the Tregs involves viral-based transfer (i.e., lentivirus or retrovirus) and although to date no safety concerns have been reported with genetically engineered T cells, using non-viral vehicles have been gaining traction, such as transposon/transposases (i.e., Sleeping beauty, *piggyBac* transposon) or gene-editing tools which will also be discussed (76). With respect to expansion, protocols already developed for polyclonal Treg infusion can be employed for CAR-Tregs. Alternatively, semi-automatic systems employed in CAR-T cell development such as rocking-motion bioreactors and static culture bags can be optimized for CAR-Treg expansion (77). The number of Tregs needed for therapy remains unclear and the doses of administered Tregs varied in different trials. We have injected polyclonal Tregs ranging from 10^5^ to 10^7^ cells/kg bodyweight in the ThRIL and the ONE Studies (17, 18) The prediction is that fewer numbers of CAR-Tregs would be needed, although solid organ transplant trials employing antigen-specific Tregs have ranged up to 9 × 10^8^ cells [for more details please refer to (9)].

## Enhancing CAR-Tregs

Engineering CAR-Tregs for clinical applications include boosting their potency, persistence, and safety. Given that CARs are composed of building blocks, modifying the scFv targeting moiety, or the intracellular co-stimulatory signaling domain has been a focus, and will be discussed herein. Additional payloads to the construct such as including safety switches or *in vivo* tracking modalities like imaging tracers are also discussed.

Like conventional T cells, Tregs express an array of different stimulatory and inhibitory receptors (78). However, the function of each of these receptors in Tregs may be different compared to conventional T cells. Due to the various properties of different co-stimulatory molecules, it is unlikely that one particular co-stimulatory molecule can serve all therapeutically required purposes for CAR-Treg therapy. Therefore, it is likely that for optimal function and persistence of therapeutic CAR-Tregs will be different, perhaps simultaneous co-stimulation signals are required, and possibly at different time points.

### Optimizing CAR-Tregs for Universal Recognition and Function

Most of the available studies in pre-clinical models of diseases have been focusing on mono-specific CAR-Tregs. Increasing the specificity of CAR-Tregs could boost their therapeutic efficacy, coined with the added advantage of Tregs functioning indirectly through bystander suppression. Different methods of implementing universal recognition of CAR-Tregs are reviewed.

**Figure 1:**
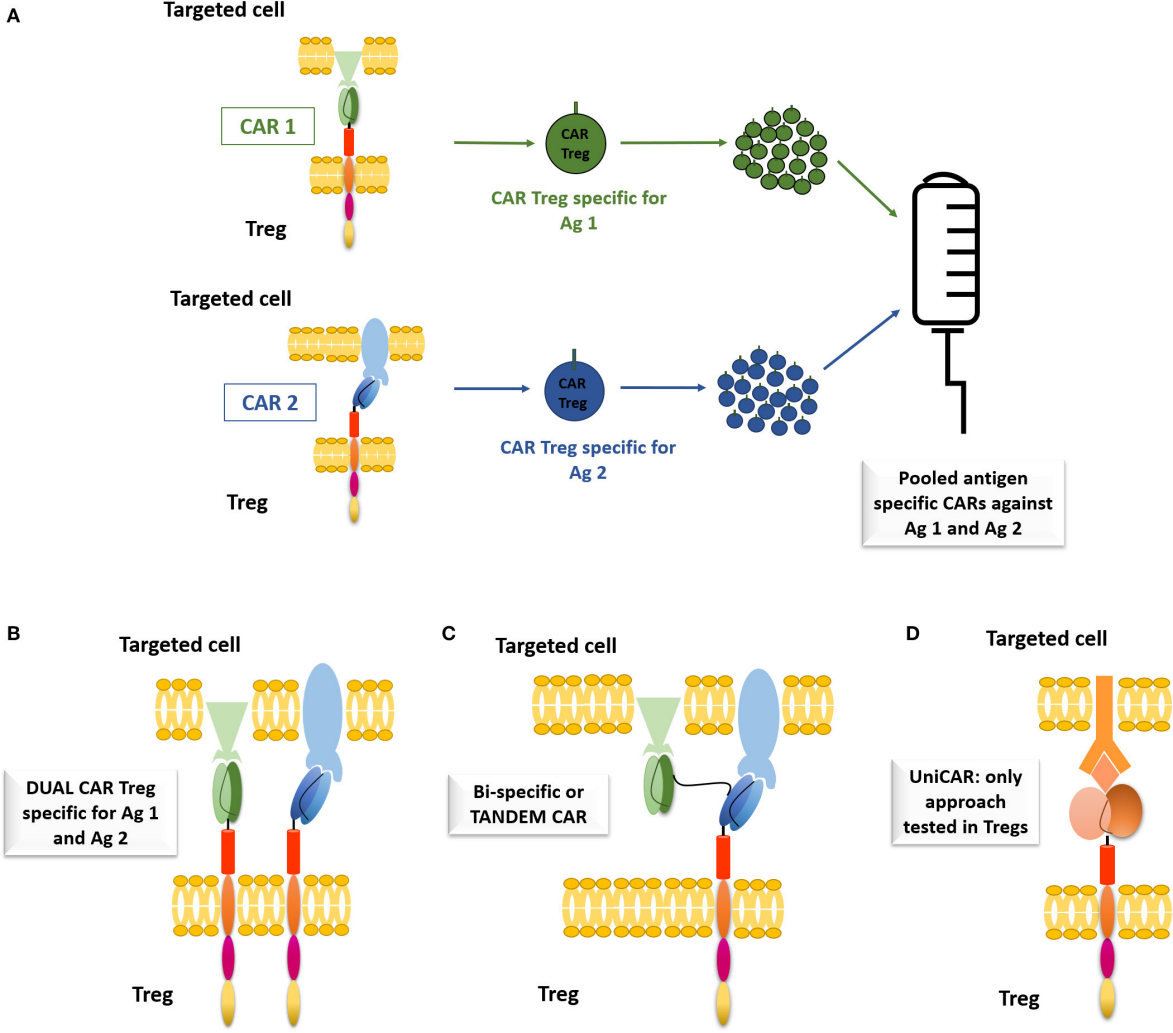
Alternatives to CAR monospecificity. **(A)** Pooled antigen specific CARs against two different antigens. CAR1 targeting Antigen 1 (Ag1), displaying an anti-Ag1 scFV, a CD28 extracellular region, a CD28 transmembrane domain, a CD28 signaling domain and a CD3ζ signaling domain, CAR2 targeting antigen 2 (Ag 2), with anti-Ag2 scFv. **(B)** Dual CAR—two different CARS connecting molecule to interact with the cell of interest. **(C)** Bi-specific or Tandem CAR—CAR able to interact with two different antigens. **(D)** Uni-CAR—using a connecting molecule to interact with the cell of interest. The CAR is displaying an anti-connecting molecule ScFv and the targeted cell has a receptor for the connecting molecule. Only approach tested with Tregs.

The first option is to infuse a pool of CAR-Tregs with different specificities (Figure 1A). This has been tested by pooling monospecific CAR-T cells targeting CD19/CD123 for B-ALL and human epidermal growth factor receptor-2 (HER2)/IL-13Rα2 for glioblastoma (79–81). However, this is logistically challenging, as expansion of autologous CAR-Tregs specific for different target antigens would be limited by the number of autologous Tregs available and the high numbers of antigens to target. Therefore, combinatorial antigen strategies or dual CAR-T cells have been developed (Figure 1B) using cells transduced with two different CARs with different antigen specificities and signaling domains (79, 80, 82, 83). The dual CAR-T cells were more efficient than pooled CAR-T cells in preventing antigen escape and demonstrated increased anti-tumor efficacy (79). Bi-specific CARs (or Tandem CAR) targeting two different antigens can also be used (Figure 1C) (81, 84, 85), but limitations include mouse scFv immunogenicity, the cross-pairing of the variable light and heavy chains between different scFvs and limited viral vector package size (86). Developing a modular or universal CAR (UniCAR; Figure 1D) where the CAR utilizes a soluble connecting molecule to engage the antigen of interest is also another strategy (87). Cells of interest are indirectly connected to the UniCAR through a distinct targeting module, called CAR-adaptors [*cf*. (88)]. Therefore, a tailored control of the Treg activity is possible, as the activation of the UniCAR-Tregs is strictly dependent of the targeting module and changing the targeting module opens to universal applications. Koristka et al. showed that Tregs derived from patients with autoimmune conditions were successfully engineered with UniCARs with 4-1BB/CD3ζ intracellular domains and these UniCAR-Tregs were able to suppress patient-derived effector cell functions, as determined by luciferase-expressing PC3-PSCA cancer cells (87). A FITC-CAR-Treg has been described by Pierini et al., which allows the combination of any monoclonal antibody to the FITC-CAR, facilitating a customisable approach to targeting antigens. The efficacy of the FITC-CAR-Tregs was demonstrated by showing that the injection of H-2D^d^-mAbCAR-Tregs into B6 mice increased the survival of BALB/c skin and islet allograft as compared to isotype-mAbCAR-Tregs (89). These last approaches are quite promising and it is the first step toward off-the shelf therapies, which could help improving the deliverability and cost associated with these treatments.

### CAR Co-stimulatory Endodomain Function in Tregs

Different co-stimulatory molecules provide different functions. Thus, it is unlikely that one particular co-stimulatory molecule can serve all therapeutic purposes required for CAR-Treg therapy. Therefore, it is likely that for optimal function and persistence of therapeutic CAR-Tregs, a particular co-stimulatory endodomain is chosen and used for the disease or health indication context that best benefits from this co-stimulatory endodomain. In addition, perhaps a combination of co-stimulation signals are required, and possibly at different time points to achieve a robust or efficient CAR-Treg therapy for patients.

The importance of the CD28 co-stimulatory domain in CAR-Tregs has been demonstrated by various groups. MacDonald et al. demonstrated that alloantigen-specific HLA-A2-specific CD28 CAR-Tregs were superior to non-targeted CAR-Tregs at preventing xenogeneic Graft vs. Host disease (GvHD) (50). We used a CAR specific for HLA-A2 that did not have an endodomain signaling component (ΔCAR) but still contained the targeting domain (i.e., ScFV specific for HLA-A2) and showed that although they were less efficient compared to fully functional CD28 CAR-Tregs *in vitro*, in a humanized mouse model of human skin transplant, ΔCAR-Tregs offered greater graft protection than polyclonal Tregs but less than CD28 CAR-Tregs. We concluded that CAR-Treg localization and activation via the TCR are important feature for their immunosuppressive capacity (51). Similarly, Noyan et al. showed that HLA-A2 specific CD28 CAR-Tregs prevented skin allograft rejection in a human skin transplant mouse model (52).

However, other co-stimulatory molecules expressed by Tregs could potentially enhance their function, stability (avoiding conversion to effector cells) and survival. To this end, Boroughs et al. performed a side-by-side comparison of CAR-Tregs expressing CARs encoding CD28 or 4-1BB endodomains. They found that CAR-Tregs with the CD28 endodomain maintained their inhibitory function whereas CAR-Tregs with the 4-1BB endodomain did not. Furthermore, only CD28 CAR-Tregs and not 4-1BB CAR-Tregs were effective suppressors of T-effector cells *in vivo* and were the most effective at inhibiting EGFR-CAR Teff mediated damage on EGFR^+^ skin transplant (90). This is in contrast with what has been published with CAR-T cells, in which the 4-1BB endodomain but not the CD28 endodomain reduced CAR-T cell exhaustion resulting in enhanced CAR-T cell persistence and longevity (57, 91). Despite several recent successes, the overall understanding of the mechanisms governing Treg stimulation remains somewhat limited. While the evidence base is rapidly increasing, more work will be required to gain insight into these precise mechanisms to generate optimized potent and long-term stable therapeutic Tregs.

## Engineering Beyond the CAR

### Enhancing the Safety Profile of CAR-Tregs

In clinical trials patient safety is of the highest priority. With cellular therapy at the clinical trial phase it is not certain whether the therapeutic cells will reach their intended destination within the patient's body and thus off-target effects may occur (92–94). In the context of CAR-Tregs, if it were to function off-target it could ensue a situation in which the patient experiences pan-immunosuppression which leads to a reduced appropriate immune response against opportunistic infections and possibly cancer development. One way to control the life of these injected therapeutic cells in the patient is to include a suicide gene feature within these genetically modified therapeutic cells before injecting them back into the patient. Suicide genes are like a “safety switch” that permits selective death on expressing cells in the event of elevated toxicity by administration of an activating soluble pharmaceutical agent in the patient (92–94). Examples of suicide genes include surface proteins such as RQR8 (93) and huEGFRt (92) which can be recognized by monoclonal antibodies (mAbs). A potential drawback of mAbs-mediated suicide genes is that the concentrations required for efficient elimination may not be easily achieved due to accessibility of the mAbs to desired tissues.

Other suicide genes can be activated by small molecules such as the herpes simplex virus thymidine kinase (HSV1-*tk*) and inducible caspase 9 (iCasp9) systems (94). HSV1-*tk* is a non-toxic enzymatic protein that converts pyrimidine and acycloguanosine nucleoside analogs for example ganciclovir into phosphorylated compounds that are toxic metabolites that presents as chain terminators and specifically kill transduced cells. This technology is widely used for cancer therapy (95). iCasp9 is a fusion of a modified human FK506 binding protein-12 (FKBP12) with the catalytic domain of human caspase 9, and its conditional dimerization allows for its activity. iCasp9 has low potential immunogenicity and its function upon activation is specific to the transduced cells. Furthermore, iCasp9 maintains function in T cells overexpressing anti-apoptotic molecules (94). These properties could promote the choice for iCasp9 as a safety feature element in CAR-Tregs amongst other human T cell therapies. Di Stasi et al. published a study which enrolled five patients who had undergone stem-cell transplant for relapsed acute leukemia and treated with iCasp9-expressing T cells. With a single dose of the dimerising drug it eliminated more than 90% of the iCasp9-expressing T cells (96). The iCasp9 safety switch has been incorporated in second generation CAR-T cells used in clinical trials targeting GD2 for cancer treatment (NCT01822652, NCT02439788) (59). Another clinical trial using fourth generation CAR-T cell therapy also employed the iCasp9 technology (NCT02992210) (59).

Overall, it could be envisioned that the ideal CAR-Treg product would be armored with an array of efficient co-stimulatory domains and suicide genes.

### Reporters for Spatiotemporal *in vivo* Tracking

The administration of live cell therapeutics including CAR-Tregs raises several important questions pertaining to cell therapy localization and relocalization over time, sites of activity and overall fate of administered cells. The existence of adoptively transferred cells can be demonstrated with highly sensitive methods based on blood samples. Cytotoxic T-cells have been shown by qPCR to be present years after administration in some patients (97). Administered Tregs have also been demonstrated to be present for a long time in the circulation of patients using a stable isotope labeling approach based on deuterium; polyclonal Tregs labeled with [6,6-^2^H_2_]glucose were detected in the circulation of Type I diabetes patients for up to 1 year (14). Importantly, these methodologies suffer from not providing answers to questions relating to spatial localization, activity, and fate of the therapeutic cells at target sites. Non-invasive whole-body imaging would be a highly beneficial tool to answer all these questions in a spatiotemporal manner.

The field of *in vivo* cell tracking has re-gained new momentum through the development of adoptive cell therapies. The various cell tracking methodologies including a variety of experimental design considerations and caveats have recently been comprehensively reviewed (98), also in the context of tracking T cell therapies (99). Fundamentally, cells require labeling to visualize them *in vivo* using technologies with exquisite sensitivities. Non-invasive radionuclide imaging by single photon emission computed tomography (SPECT) or positron emission tomography (PET) offers excellent sensitivity with absolute quantification and true 3D information while being translatable to the clinic. Labels can be introduced into cells via two fundamentally different methodologies, direct and indirect cell labeling (98).

So-called “direct cell labeling” employs ready-to-use contrast agents (e.g., organic fluorophores, quantum dots, iron oxide nanoparticles, ^19^F-fluorinated contrast agents, chelated radiometals etc.), which are introduced into cells either due to the contrast agents being cell permeant, or through assisted uptake (e.g., by transfection or internalization). We previously showed that direct radiolabeling of polyclonal murine CD4^+^ T cells with ^99m^Tc-hexamethylpropyleneamine oxime did not affect cell viability, but the radiolabeled cells could only be tracked for up to 24 h due to the short half-life of the radiolabel [half-life of ^99m^Tc is 6.01h; (100)]. This enabled the assessment of Treg biodistribution within a day of administration but precluded long-term tracking of Tregs. Longer half-life isotopes could provide this opportunity, albeit are not free of caveats. The SPECT isotope ^111^In has been used clinically to follow directly labeled white blood cells for decades (101), but due to its decay properties it has also been associated with significant radiodamage (102). ^89^Zr has a similar half-life as ^111^In and was used to track cells for up to 2 weeks (103, 104). With clinical PET being more sensitive than SPECT, not least through the very recent development of total-body PET, which has been shown to be another 40-times more sensitive than conventional PET (105), ^89^Zr-labeling would result in the use of less radioactivity to achieve the same tracking results. However, radio-damage as a consequence of radioisotope incorporation into cells must be assessed, particularly in cell types such as T cells that are routinely ablated using radiation. Therefore, careful dosimetry considerations are required to assess both the preclinical and clinical feasibility of Treg tracking via this route [for caveats see (98)].

The alternative is “indirect cell labeling,” whereby a genetically encoded reporter is ectopically introduced into the cells mostly by viral transduction to ensure genomic integration and thus stable long-term expression; transposon and gene editing represent alternative methodologies (106, 107). Reporter genes have critical advantages over direct labeling for cell tracking (99, 108). First, the observation period is independent of the contrast agent, for example, not affected by the half-life of a radioisotope. Second, genetic encoding avoids label dilution phenomena, which are limiting observation times in the case of fast-growing cells (e.g., expanding T cells). Third, genetic encoding circumvents complex direct cell labeling procedures and potential associated cell toxicities. A drawback of the indirect cell labeling approach is that it requires genetic engineering. However, this is not a concern for preclinical experimentation and not a concern for adoptive cell therapies that require genetic engineering to confer targeting specificity and/or efficacy, such as CAR-Treg therapy. Treg *in vivo* dynamics has been assessed preclinically using bioluminescence reporters (109, 110). Dawson et al. tracked HLA-A2 CAR-Tregs *in vivo* using bioluminescence and found that the peak of CAR-Treg infiltration to A2^+^ skin graft was 7 days post infusion (53). However, this imaging modality is not clinically translatable because of the non-human nature of luciferases, and the added disadvantages of optical imaging at depth (absorption, scatter) precluding reliable quantification. As foreign reporters can elicit an immune response and result in immune destruction of the administered therapeutic cells, a host-compatible reporter is preferable in this context. Host reporters are from the same species but endogenously expressed in only a very limited number of host tissues, and ideally at low levels to ensure favorable contrast (99). The most promising host reporters available for the purpose of Treg tracking in skin transplant models are the human sodium iodide symporter (NIS) (111) and the human prostate-specific membrane antigen (PSMA) (112), as neither of them is expressed in human or mouse dermis or epidermis. NIS offers the advantage of a generator-produced radiotracer ([^99m^Tc]${\text{TcO}}_{4}^{-}$) for SPECT imaging avoiding complex synthesis on each imaging day. Notably, there is also a clinical PET tracer available for NIS ([^18^F]${\text{BF}}_{4}^{-}$), which is accessible via an automated synthesis protocol (113, 114). Notably, Volpe et al. have also demonstrated that NIS expression and use for imaging did not result in radiodamage-related negative effects in CAR-T cells (115). In a proof-of-principle study employing retroviral transduction methodology, we demonstrated *ex vivo* engineering of murine Tregs to express a radionuclide imaging reporter and detected them 24 h post administration by SPECT imaging (116). However, so far long-term tracking of human Tregs has not been addressed using clinically translatable imaging technologies and remains an important area of future research to aid the development and clinical translation of adoptive Treg therapy.

## Translating CAR-Tregs to the Clinic

The quick evolution of CAR-T cells into clinic has informed the scientific community of the pitfalls and hurdles associated with delivering an effective, safe and reproducible treatment; applying the lessons to CAR-Treg therapy should accelerate their use in clinic. Fritsche et al. extensively reviewed optimized methods in manufacturing GMP-grade CAR-Tregs (117) but factors including generating “off-the-shelf” products, increasing *in vivo* persistence and eliminating CAR-associated toxicities are a few examples of hurdles to overcome and will be discussed next.

Developing next-generation, or “off-the-shelf,” products is a focal point for clinical translation of CAR-T cells and, equally, must be considered for CAR-Treg therapy. Currently, the manufacturing process of autologous CAR-T cells for cancer patients incur a few paramount disadvantages, such as possible failure during manufacturing, and critically, the 3 week long process of developing the treatment which is a setback in highly proliferative malignancies [reviewed in (118)]. Time critical treatment delivery is not as big of a concern for CAR-Tregs in autoimmunity and solid organ transplant rejection. However, risk of failure due to low absolute numbers or functionally defective Tregs because of the disease, or interference from adjunct immunosuppressive medications need to be considered. Most importantly, the high cost incurred of manufacturing and delivery patient derived CAR-T cells has been a challenge for health care systems and needs to be considered if CAR-Treg therapy is to be translated into clinic. Previously, allogeneic CAR-T cells generated from “healthy donors” have been considered as a fast, scaled-up and decreased cost method of which high numbers of CAR-T cells can be produced per donor, with the added advantage of cryopreserving large batches, ready for treatment immediately. However, this gave rise to GvHD or clearance by the host's immune system (119). Different strategies have looked at generating manipulated “off the shelf” CAR-T cell products.

The use of gene editing as a tool for generating off the shelf CAR-T cells is very promising and can be translated to CAR-Treg therapy. This can be achieved by using transcription-activator-like effector nucleases (TALENs) to knock out the TCRα chain (TRAC) or β2 microglobulin of the MHC molecule, to prevent alloreactive T cells from inducing GvHD (120). CRISPR-Cas9 is another tool to replace the TCRαβ with the CAR in the TRAC locus or β2 microglobulin of the MHC molecule to minimize immunogenicity avoiding GvHD (121, 122).

Concerns surrounding candidate patients who are on immunosuppressive regiments may also interfere or crosstalk with CAR-Treg efficacy. Drug such as antithymocyte globulin (ATG), cyclosporin, anti-CD25 and rapamycin are administered to transplant recipients and have an impact on Treg numbers and function. ATG reduces the absolute number of Tregs and high doses has been linked to impaired thymic Treg development in allogeneic HSCT (123). Cyclosporin and other calcineurin inhibitors (e.g., tacrolimus) suppress Treg activation and decrease FOXP3 expression but this can be restored by administration of IL-2 (124). We have shown in the ThRIL study the efficacy of Treg therapy in patients on immunosuppressive regimens including ATG and tacrolimus, which is encouraging for future CAR-Treg trials (17). In contrast, drugs such as sirolimus or everolimus, (rapamycin) inhibitors of the mTOR pathway may have a beneficial effect as used in combination with Tregs in the treatment of transplant patients as rapamycin is routinely used in the *ex vivo* expansion of Tregs and promote Tconv outgrowth (125).

## Concluding Remarks

CAR-Tregs are the logical extension of polyclonal Treg therapy to enhance their efficacy by conferring antigen-specificity. It is an emerging area with not an insignificant amount of research required to develop and adapt existing CAR-Treg concepts and optimize them for successful clinical translation. As reviewed here the application of CAR-Tregs to the clinic needs further refinement. There is a need to maximize their suppressive function, their stability and understand better their homing capacity and longevity i.e., preventing CAR-Treg exhaustion. Such cell products raise another concern and this is the cost (126). Currently, treating a patient with anti-cancer CAR-T cell therapy costs $400,000 without the ancillary costs (127). Furthermore, the critical rate needed to manufacture personalized products, the failure to achieve the targeted cell numbers in some patients, and the heterogeneity of the cell products generated need to be overcome. However, the safety demonstrated with the clinical application of polyclonal Tregs and the pre-clinical data with CAR-Tregs has now generated investment in CAR-Treg therapy and several start-up companies have been funded, with the aim of applying CAR-Tregs to cure autoimmune diseases and induce transplantation tolerance. The first CAR-Treg clinical trial has been granted by UK MHRA authorization in a phase I/II clinical trial (STEADFAST) for kidney transplant patients. Progress in our understanding of the biology of Tregs, the ability of functional enhancements through genetic engineering, contribute to the excitement of this field of research.

## Author Contributions

YM participated in manuscript writing, editing and coordination of its submission. ST and CD contributed to manuscript writing. RL, GF, and GL contributed to manuscript writing and editing. All authors contributed to the article and approved the submitted version.

## Conflict of Interest

The authors declare that YM and ST are employed by Quell Therapeutics. GL is co-Founder of Quell Therapeutics. The remaining authors declare that the research was conducted in the absence of any commercial or financial relationships that could be construed as a potential conflict of interest.

## Abbreviations

A^b^: mouse MHC Class II molecule.
Aurora B kinase: a protein involved in the cell cycle; it functions in attaching the mitotic spindle to the centromere.
Bcl-2 (B-cell lymphoma 2): a protein, which regulates programmed cell death; anti-apoptotic protein that functions by preventing mitochondrial apoptogenic factors such as cytochrome c and apoptosis-inducing factor (AIF) to be released into the cytoplasm.
Bcl-xL (B-cell lymphoma-extra large): a protein that regulates programmed cell death in a similar manner as Bcl-2.
GvHD (Graft-vs.-host disease): an immune condition that occurs after transplant procedures when immune cells from the donor (known as the graft or graft cells) attack the recipient patient host's tissues; the disease is a side effect that is common after an allogeneic bone marrow transplant (stem cell transplant).
CRISPR/Cas9: (the clustered regularly interspaced short palindromic repeats system): combines a nuclease and a short RNA; specificity depends on RNA-DNA base pairing whereby the RNA is complementary to the genomic target DNA. The system most commonly uses Cas9, delivering the nuclease to the target site.
HuEGFRt: a human epidermal growth factor receptor (EGFR) polypeptide synthesized for cell selection by binding of an anti-EGFR monoclonal antibody.
Human FK506 binding protein-12: a 12kD cytosolic protein expressed ubiquitously; functions as a molecular chaperone for protein folding.
Immunodysregulation polyendocrinopathy enteropathy X-linked (also known as IPEX): a rare disease associated with FOXP3 dysfunction, leading to Treg impairment and severe autoimmunity.
PSMA (Prostate-specific membrane antigen): a protein specifically expressed in prostate tissue carcinomas derived from it.
RQR8: the protein product of a suicide gene; more speciofically, a 136 amino acid construct, which enables selection with the cliniMACS CD34 system and *in vivo* depletion of the administered cells with rituximab.
Scurfy mouse: due to a mutation in the Foxp3 transcription factor, Scurfy mice lack regulatory T-cells that maintain self-tolerance of the immune system.
PET (Positron Emission Tomography): a radionuclide 3D imaging modality used in the clinic; suitable radionuclides are incorporated into radiopharmaceuticals, which are then used to image specific biological process *in vivo*. The radioisotope must be a positron emitter, whose emitted positrons combine with electrons to produce two gamma rays pointing into opposite directions; these gamma rays are detected by the instrument and via reconstruction algorithms a 3D image is formed.
SPECT (Single Photon Emission Computed Tomography): a radionuclide 3D imaging modality used in the clinic; radionuclides are incorporates into radiopharmaceuticals, which are then used to image specific biological processes. The radioisotope must be a suitable gamma ray emitter; gamma rays pass through a collimator and are detected prior to econstruction and 3D image formation.
Survivin: a protein that regulates apoptosis and the cell cycle; it functions alongside aurora B kinase in facilitating completion of the cell cycle. Survivin forms a chromosomal passenger complex that regulates chromosome-microtubule attachment, proper spindle assembly and occurrence.
TSDR (Treg-specific demethylated region): an evolutionarily conserved element within the FOXP3 locus.
